# Large-Scale Computational Screening Identifies First in Class Multitarget Inhibitor of EGFR Kinase and BRD4

**DOI:** 10.1038/srep16924

**Published:** 2015-11-24

**Authors:** Bryce K. Allen, Saurabh Mehta, Stewart W. J. Ember, Ernst Schonbrunn, Nagi Ayad, Stephan C. Schürer

**Affiliations:** 1 Department of Molecular and Cellular Pharmacology, Miller School of Medicine, University of Miami, Miami, FL, US; 2 Center for Computational Science, University of Miami, Miami, FL, US; 3 Department of Applied Chemistry, Delhi Technological University, Delhi, India; 4 Drug Discovery Department, H. Lee Moffitt Cancer Center and Research Institute, Tampa, FL, US; 5 Center for Therapeutic Innovation Miller School of Medicine, University of Miami, Miami, FL, US; 6 Miami Project to Cure Paralysis, Department of Psychiatry and Behavioral Sciences, Miller School of Medicine, University of Miami, Miami, FL, US

## Abstract

Inhibition of cancer-promoting kinases is an established therapeutic strategy for the treatment of many cancers, although resistance to kinase inhibitors is common. One way to overcome resistance is to target orthogonal cancer-promoting pathways. Bromo and Extra-Terminal (BET) domain proteins, which belong to the family of epigenetic readers, have recently emerged as promising therapeutic targets in multiple cancers. The development of multitarget drugs that inhibit kinase and BET proteins therefore may be a promising strategy to overcome tumor resistance and prolong therapeutic efficacy in the clinic. We developed a general computational screening approach to identify novel dual kinase/bromodomain inhibitors from millions of commercially available small molecules. Our method integrated machine learning using big datasets of kinase inhibitors and structure-based drug design. Here we describe the computational methodology, including validation and characterization of our models and their application and integration into a scalable virtual screening pipeline. We screened over 6 million commercially available compounds and selected 24 for testing in BRD4 and EGFR biochemical assays. We identified several novel BRD4 inhibitors, among them a first in class dual EGFR-BRD4 inhibitor. Our studies suggest that this computational screening approach may be broadly applicable for identifying dual kinase/BET inhibitors with potential for treating various cancers.

Kinase inhibitors have been identified for the treatment of various cancers^1^,^2^. However, compensatory mechanisms diminish the long-term efficacy of these inhibitors^3^. Drug resistance is often observed in the clinic as rapidly dividing cancer cells are able to avoid inhibition by a single targeted therapy through a variety of mechanisms^4^. The resistance of tumors toward kinase-directed therapeutics is often accompanied by a distinct change in signaling network composition through adaptive kinome reprogramming, allowing the tumor to elude effects of the drug and manifest resistance^5^. An established strategy to improve the durability of clinical responses to targeted therapies is to simultaneously inhibit multiple cancer-driving kinases. However, discovering kinase inhibitors with an appropriate multitarget profile has been challenging and necessitated the application of combination therapies, which can pose major clinical development challenges^6^,^7^,^8^,^9^. We therefore sought a strategy to identify single agent polypharmacological compounds with the ability to target multiple cancer promoting pathways, but that does not rely on inhibiting multiple kinases. We chose to target epidermal growth factor receptor (EGFR) along with the epigenetic reader bromodomain-containing protein 4 (BRD4). EGFR is a receptor tyrosine kinase (RTK) that is amplified or mutated in several cancers and is the subject of intensive drug discovery efforts^10^,^11^,^12^. Similarly, BET bromodomain proteins have recently emerged as possible drug targets in multiple cancers. BET proteins are epigenetic readers that primarily recognize acetylated lysine residues on histones, and function in regulating gene transcription^13^. Their role in modulating chromatin structure is important for proper cellular function and expression of genes involved in multiple signaling pathways. BET proteins have been implicated in cancer cell proliferation by controlling the activity of various oncogenes required for cell cycle progression^14^. BRD4 is possibly the best-characterized BET protein, which contains two regions that bind acetylated lysine residues termed bromodomains, Bromodomain 1 (BRD4(1)) and Bromodomain 2 (BRD4(2)). Both domains bind to acetylated histones primarily through interactions in the ZA loop and BC loop-helix junctions of BRD4(1) and BRD4(2)^15^. Highly selective small molecules are able to displace these bromodomains from chromatin; thereby reducing transcription of oncogenes, such as MYC. Several small molecule BRD4 inhibitors have been developed, which show efficacy in reducing growth of multiple tumors *in vivo* and are in clinical trials for the treatment of solid tumors^16^,^17^. Thus, BRD4 is a promising drug target for the treatment of various cancers. Interestingly, some known kinase inhibitors potently inhibit BRD4, suggesting that the therapeutic efficacy of these compounds may be due in part to BRD4 inhibition^18^,^19^. In addition, use of the BRD4 inhibitor JQ1 in combination with the EGFR inhibitor lapatinib has been shown to suppress lapatinib-induced kinome reprogramming in ERBB2+ breast cancer cells, where other kinase inhibitor combinations could not^5^. This knowledge-based rationale is also supported by data from the Library of Integrated Network-based Cellular Signatures (LINCS, http://www.lincsproject.org/). We show that transcriptional response signatures of known EGFR and BRD4 compounds are distinct from one another as well as from a background population, suggesting that EGFR and BRD4 inhibitors utilize orthogonal signaling networks and different transcription factors, therefore supporting the idea of prolonged efficacy and reduced resistance when using a compound that targets both proteins. To identify such dual inhibitors we describe a large-scale computational screening pipeline, which leads to the discovery of novel BRD4 inhibitors and a first in class multitarget EGFR and BRD4 inhibitor. We suggest that this virtual screening protocol can be adopted across the human Kinome for identifying dual kinase-BRD4 inhibitors.

## Results

### Transcriptional profiles of EGFR and BRD4 inhibitors show distinct signatures

**Figure 1:**
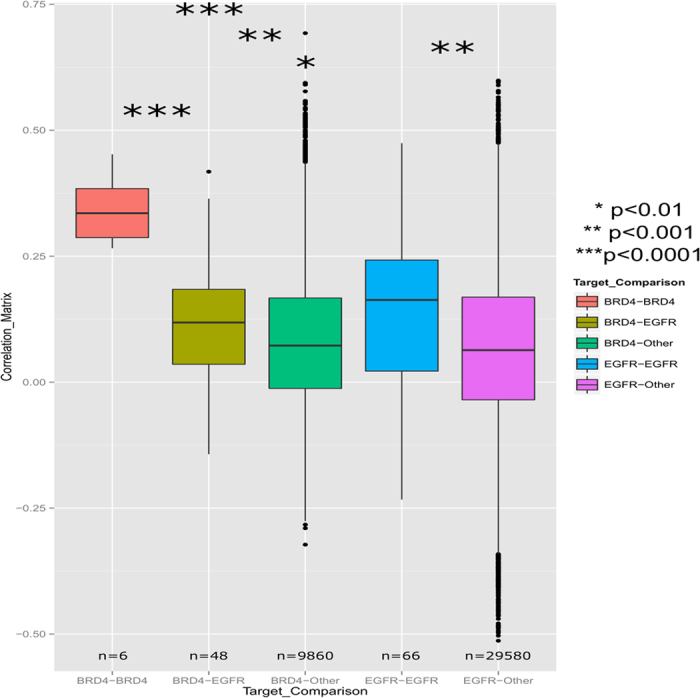
Transcriptional profiles of EGFR and BRD4 inhibitors are distinct. The box plot shows correlation matrix variances for each comparison of signatures generated by target inhibitors in the MCF7 cell line at 10 *μ*M for 24 hours. P-values obtained by Welch’s two sample t-test comparing the populations of pairwise correlations.

The Library of Integrated Network-based Cellular Signatures (LINCS) program (http://www.lincsproject.org) is producing large profiling datasets and computational tools to advance the development of systems-wide network-based disease models with the goal to develop more efficacious and safer therapeutics. LINCS datasets, for example, include genome-wide transcriptional profiles across a wide range of cell lines and tens of thousands of drug and genetic perturbations generated at the Broad Institute (http://www.lincscloud.org/). We have previously shown how transcriptional profiles correlate to chemical similarity, LINCS (KINOMEScan) and predicted small molecule kinase activity as well as their enrichment by signaling pathways^20^. Here we demonstrate that transcriptional signatures of EGFR kinase and BRD4 inhibitors are distinct from one another as well as from other perturbation profiles. This would be expected if the cellular response to these inhibitors occurs via orthogonal pathways and transcription factors. We used data generated via the L1000 assay (http://www.lincscloud.org/l1000) in MCF7 cells. Twelve reported EGFR inhibitors and four known BRD4 inhibitors (Supplementary Table 1) were included in the dataset. Pearson correlation coefficients were computed for all pairwise signatures. Four main signature populations, namely, only EGFR, only BRD4, the intersection of EGFR and BRD4, and all Other inhibitors, were compared using Welch’s two sample t-test (Fig. 1). Highly significant p-values in the range of 10^−5^ to 10^−4^ were obtained when comparing EGFR to BRD4, BRD4 or EGFR to Other, as well the EGFR-BRD4 intersection to Other. P-values of 10^−3^ were obtained when comparing the EGFR-BRD4 intersection to either BRD4 or EGFR. These results show that signatures of EGFR and BRD4 inhibitors as a group are distinct from the global reference population (Other) as well as distinct from one another. This analysis supports our rationale of developing dual EGFR-BRD4 inhibitors.

### Dual target screening approach

**Figure 2:**
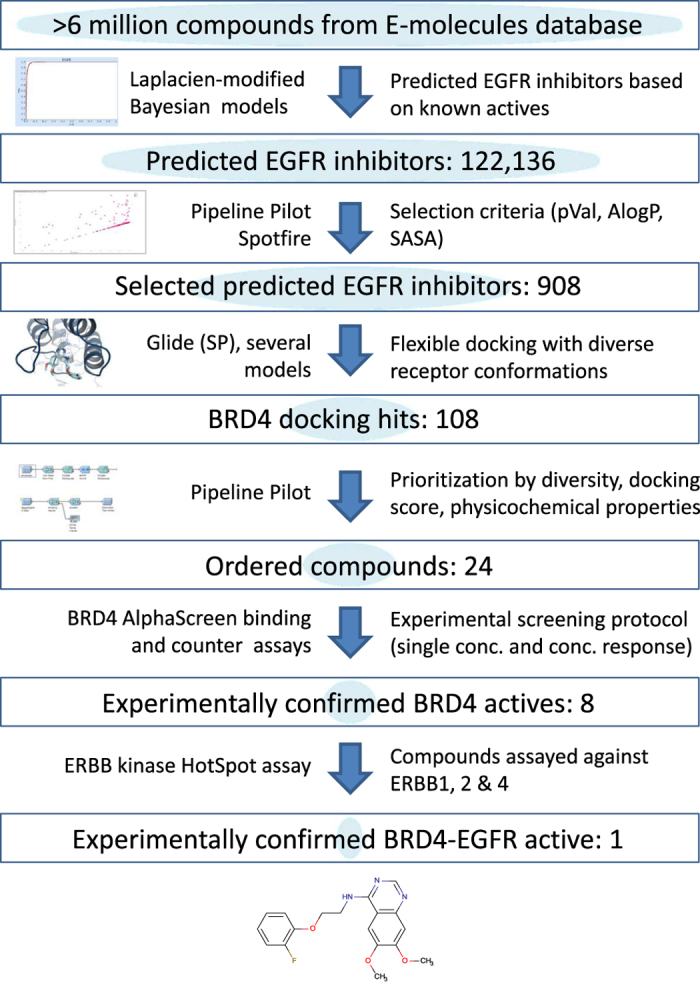
High-throughput computational screening pipeline to identify dual inhibitors of EGFR kinase and BRD4(1).

We then developed a practical computational screening protocol in order to identify dual EGFR-BRD4 inhibitors. Our virtual screening approach incorporated highly predictive ligand and structure-based models that were constructed from small molecule kinase inhibition datasets, BRD4 protein structures, and physicochemical property predictors. We successfully used this pipeline to identify novel BRD4 inhibitors and a dual EGFR-BRD4 inhibitor from over six million commercially available compounds (Fig. 2).

### Scalable machine learning classifier to predict novel EGFR inhibitors

Over three thousand unique compounds with reported EGFR kinase inhibition (IC_50_ or K_*i*_ ≤ 100 nM and close to five thousand compounds ≤1 *μ*M) are available in the Kinase Knowledgebase (KKB) (see Methods), along with almost six hundred thousand kinase inhibitors curated from articles and patents that have no reported EGFR activity (presumed inactive). Given these large datasets, we chose ligand-based Laplacien-modified Naïve Baysian classifiers that we had previously developed and described in detail^21^. The models were systematically cross-validated generating enrichment factors and representative receiver operating characteristic (ROC) curves using 50% of the data as test set (Supplementary Figure 1). Averaging 20 cross validation runs with randomly selected 50/50 split training/test compounds, ROC scores of 0.99 and 0.98 were obtained for the 1 and 0.1 *μ*M models, respectively. Enrichment factors of 78 and 66 were obtained for the top 1% of compounds, which is close to the upper bound maximum possible enrichment under these conditions. Both models were applied to evaluate over six million commercially available compounds from the eMolecules database. In addition to the standard Laplacien-modified Bayesian scores and the binary class predictions, we computed for all compounds the estimated probabilities that a sample was in the active class (EstPGood). EstPGood is based on assumed normal distribution of scores in the active and inactive class and is a normalized score that is comparable across models. We also computed basic physicochemical properties including molecular weight, hydrogen bond donor and acceptor counts, polar solvent accessible surface area, and Ghose Crippen octanol-water partition coefficient (ALogP). From the library of more than 6 million compounds with kinase activity predictions, we selected only compounds that were in the active class for both EGFR models (cutoff at 1 and 0.1 *μ*M activity). The remaining 122,136 compounds were then further filtered to simultaneously optimize probability of kinase activity and favorable physicochemical properties using interactive visual analysis and selection in TIBCO Spotfire (see Methods for details). 908 compounds were selected sampling the most likely EGFR actives based on predicted probability of EGFR activity (EstPGood), and compounds of favorable (drug-like) physicochemical properties relaxing probability of EGFR activity.

### Ensemble docking with data fusion to predict novel BRD4 binders

In contrast to EGFR kinase, publicly available small molecule inhibition and binding data for BRD4 are limited. However, co-crystal structures of BRD4 are available in the Protein Data Bank (PDB) allowing for an unbiased structure-based approach to predict BRD4 bromodomain binding. We built nine distinct docking models after selecting representative co-crystal structures, considering co-crystal ligand chemical diversity, quality and resolution of the structures and conserved water molecules that participate in ligand binding (water bridges). These structures, representing an ensemble of conformations and binding interactions of BRD4(1), were prepared, optimized, and docking models were generated and validated as described in Methods. Re-docking of the co-crystal ligands into their corresponding receptors reproduced the co-crystal poses in each case with route-mean-square deviation (RMSD) values of less than 1 Å. Prior to docking, 2D representations of compounds were processed to generate stable tautomers and protonation states at pH 7 ± 2 and 3D conformations. Compound docking was performed for each model using Glide standard precision (SP), which was run distributed over several compute nodes (see Methods). Glide is a flexible docking method to predict multiple ligand binding poses and assigns a score to each pose by estimating binding affinity, incorporating several energetic terms and empirical parameterization^22^,^23^. To maximize accuracy of the ensemble docking approach, we calculated 18 fused scores by hierarchical aggregation of the individual docking scores at three stages: (i) pose of each unique ligand representation, (ii) compound structure (tautomer and protonation state), and (iii) BRD4 structure representation (docking model) as described in detail in Methods (Supplementary Figure 2). We characterized these fusion scores by their statistical distributions, correlation to known BRD4 activities and ROC and enrichment of the resulting (fusion) models. After evaluation and considering rationale of simplicity, we chose a scoring scheme that corresponds to the best pose of the best ligand representation of each compound for the best BRD4 structure representation.

**Figure 3:**
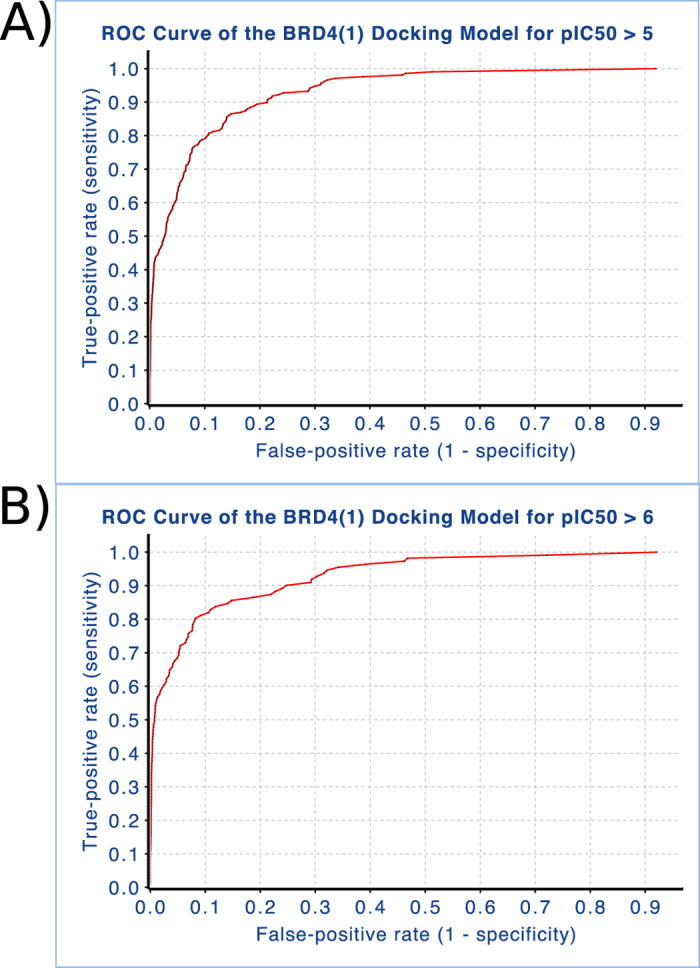
ROC curves of the BRD4 docking data fusion model. The activity cutoff is (**a**) pIC_50_ ≥ 5 and (**b**) pIC_50_ ≥ 6. The ROC curve is true positive rate (sensitivity) over false positive rate (1 - specificity).

This BRD4 data fusion model was validated using 246 known actives extracted from ChEMBL (verison 18)^24^ and curated from the literature, and 15,240 corresponding decoy compounds obtained from the Directory of Useful Decoys^25^ as described in Methods. Using the active BRD4 compounds and the decoy set, we computed sensitivity (true positive rate), specificity, accuracy, enrichment factors and ROC score of the docking fusion model. The docking score cutoffs of ≤−7 and ≤−8, (smaller is better) corresponded to approximately −2 and −3 standard deviations from the mean distribution of predicted compound docking scores. Four different scenarios were calculated for enrichment, with active BRD4 compounds defined as either pIC_50_/pK_*d*_/pK_*i*_ (pActivity) ≥ 5 or 6, with all others considered inactive, and predicted active docking score ≤−7 or −8 respectively (Table 1). Evaluating the docking model performance at a docking score threshold of ≤−7 gave high sensitivity indicating that the model was able to identify active molecules appropriately, but the models were less specific and accurate, compared to the evaluation of known and decoy compounds at docking score threshold of ≤−8. Notably, differences in activity value thresholds of pActivty ≥ 5 and 6 respectively gave little differences in model predictive performance; however, enrichment factors calculated at 0.1 and 1% of the top docked compounds were much higher at the pActivity ≥ 6 activity threshold. The receiver operating characteristic area under the curve (ROC score) was excellent for both activity cutoffs, further supporting the models’ predictive performance (Fig. 3). These statistical cross validation results confirm very high quality of the consensus docking data fusion model and the applicability for virtual screening.

**Figure 4:**
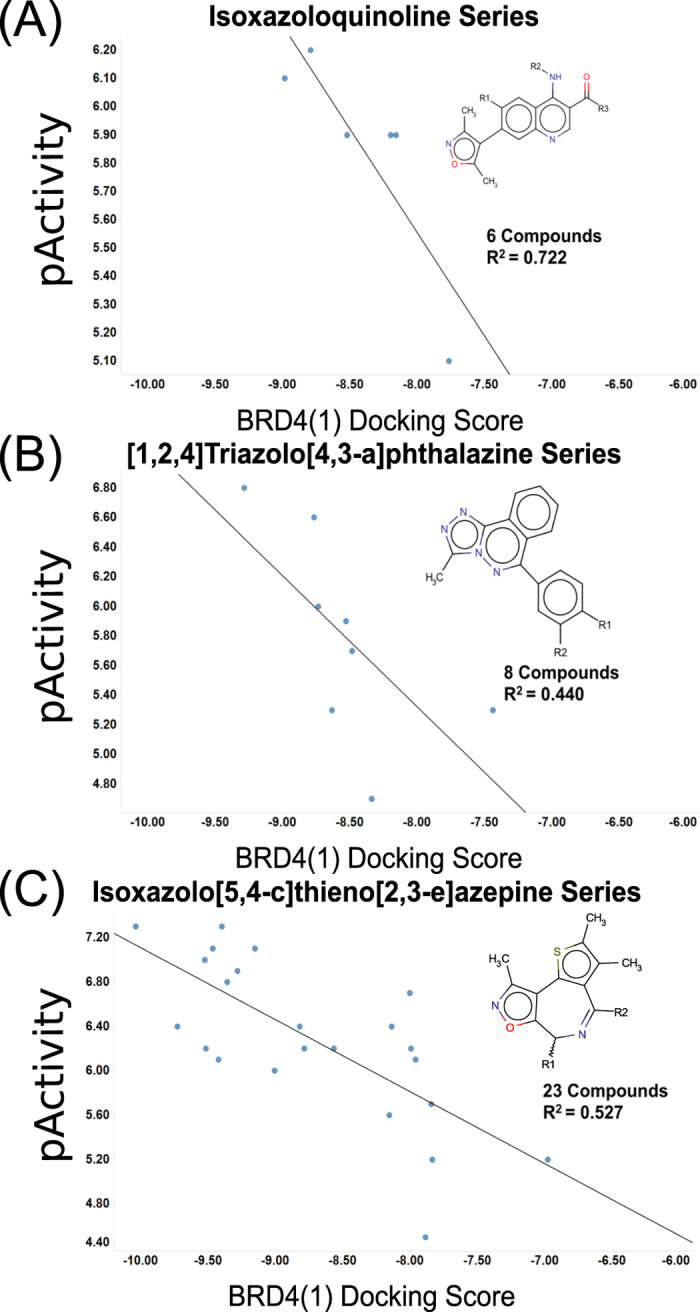
Docking scores versus reported BRD4 pActivities (pIC_50_/pK_*d*_/pK_*i*_) and Pearson correlation results of representative BRD4 inhibitors by chemotypes.

In addition to ROC scores and enrichment factors we also investigated how the aggregate docking scores and reported pActivity values relate quantitatively. As can be expected, there is no global correlation, because docking scores estimate relative binding affinity and typically cannot be compared across different binding modes. However, after clustering known BRD4 actives by maximum common substructure and topological features, we found good correlation for conserved chemotypes. Pearson correlation coefficients (R^2^) in the range of 0.4 to 0.7 were observed for three representative chemical series, namely isoxazole-substituted quinolines, triazolo-phthalazines, and isoxazolo-thieno-azepines, which include 6 to 23 compounds (Fig. 4).

We applied this BRD4(1) ensemble docking protocol to score the 908 compounds filtered from commercially available libraries using the EGFR kinase activity classifiers and physicochemical properties. Compounds were then selected based on BRD4 docking score, EGFR classifier EstPGood, commercial availability by vendor (to minimize the number of suppliers), physicochemical properties, chemical diversity and manual review. Although our docking study included known EGFR kinase inhibitors that had been selected by our classifiers from the commercial libraries, these compounds did not receive acceptable docking scores for further prioritization and testing.

### Experimental validation of predicted BRD4 and EGFR actives

**Figure 5:**
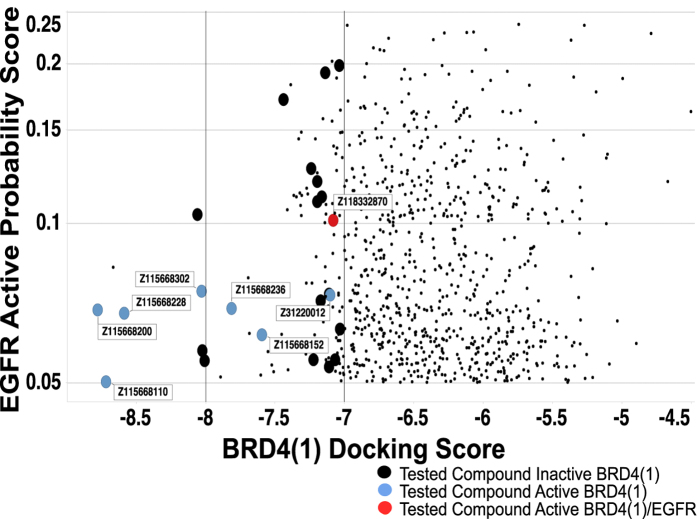
Predicted probability of EGFR activity (EstPGood for pIC_50_ ≥ 6) vs BRD4 aggregate/fusion docking score for 908 compounds that were filtered from commercial libraries using the EGFR kinase Laplacien-modified Naïve Bayes classifiers and physicochemical properties. Circles represent purchased compounds tested in the BRD4(1) assay, categorized by their activity against BRD4 and EGFR. Only compounds that were confirmed active against BRD4 were further tested for activity against EGFR; any of the black circles therefore could also be EGFR actives.

**Figure 6:**
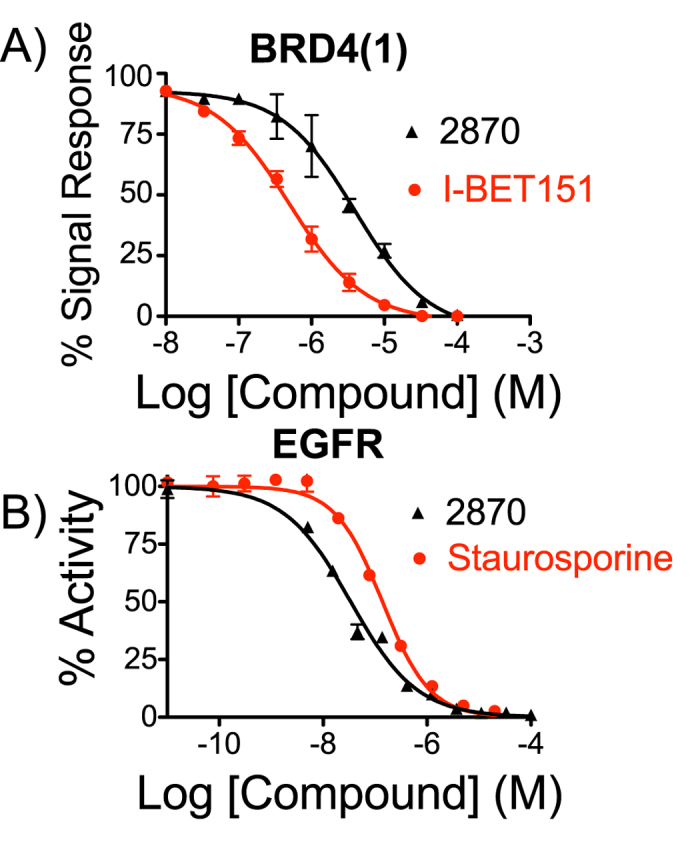
Concentration response profile of 2870 (**A**) Average concentration-response profile of compound 2870 and known BRD4 inhibitor I-BET151 using the BRD4(1) biochemical alpha screen assay (n = 3). Emission data was normalized using DMSO and is reported as % response. Average IC_50_ of 2870 was found to be 9.02 *μ*M against BRD4(1). (**B**) The EGFR kinase radioisotope filter binding assay was performed at a substrate concentration of 10 *μ*M against 2870 and Staurosporine (n = 3). Average IC_50_ of 2870 was found to be 0.044 *μ*M against EGFR kinase.

After searching multiple commercial providers for the top 108 compounds, Enamine LLC (http://www.enamine.net/) provided 24 compounds comprising 5 chemical scaffolds including substituted 2-pyrrolidinones, quinazolin-4-amines, thienopyrimidin-4-amines, and imidazolidinones (Supplementary Table 2). Figure 5 shows the 24 purchased compounds among 908 molecules that were selected using the EGFR classifiers and physicochemical properties. As illustrated, several compounds have confirmed activity against BRD4(1) and one compound, Z118332870 (2870), is active against BRD4 and EGFR kinase. Compounds that were not confirmed BRD4(1) actives were not tested in the EGFR assay because we were primarily interested in novel dual BRD4 and EGFR inhibitors. To evaluate small molecule BRD4 bromodomain binding, we utilized a biochemical alphascreen assay that measured disruption of BRD4(1) binding to pre-acetylated biotin-histone 4 peptide by proximity detection of the conjugated donor and acceptor beads via transient singlet oxygen. IC_50_ values were calculated using nonlinear regression from GraphPad Prism Version 5.0c for OSX (GraphPad Software, La Jolla California USA, www.graphpad.com) from 9 concentrations in triplicate (see Methods) with an average IC_50_ value of 9.02 *μ*M for 2870 (Fig. 6a). To validate results, a counter-screen assay (see Methods for details) was performed to ensure that compounds were not interacting with assay components other than recombinant BRD4(1) (Supplementary Figure 3). The counter-screen assay indicated that each active compound was not interfering with alphascreen assay components.

**Figure 7:**
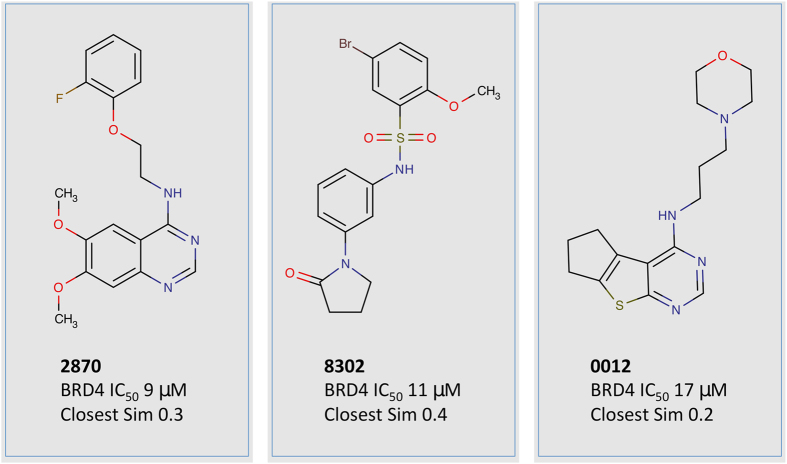
Confirmed active compounds identified by the computational screening pipeline Activity from the alphascreen assay against BRD4(1) and closest topological similarity to known BRD4(1) binders is also shown. Five other compounds comprising the same sulfonamide scaffold as 8302 were also confirmed actives but are not displayed.

Confirmed active compounds Z118332870 (2870), Z31220012 (0012), and Z115668302 (8302) represent three distinct chemotypes that are novel (by topological similarity) compared to all known BRD4 compounds (Fig. 7). Additional analogs of sulfonamide compound 8302 were also active in the alphascreen assay. To further validate the BRD4 binding affinity of 2870, differential scanning flourimetry (DSF) was performed with BRD4(1). A mean ΔTm value of 1.15 °C was obtained (Supplementary Figure 3). Based on the standard curve, this corresponds to a predicted IC_50_ value of 9.1 *μ*M^18^. We then determined whether each compound inhibited EGFR kinase (ERBB1), ERBB2 and ERBB4 using a kinase enzyme activity assay (see Methods). Only 2870 inhibited EGFR (IC_50_ = 44.2 nM) (Fig. 6b) with about 200 fold selectivity over ERBB2 (8.73 *μ*M), and 500 fold selectivity over ERBB4 (24.2 *μ*M) (Table 2).

### Characterization of EGFR-BRD4 dual inhibitors by molecular dynamics

**Figure 8:**
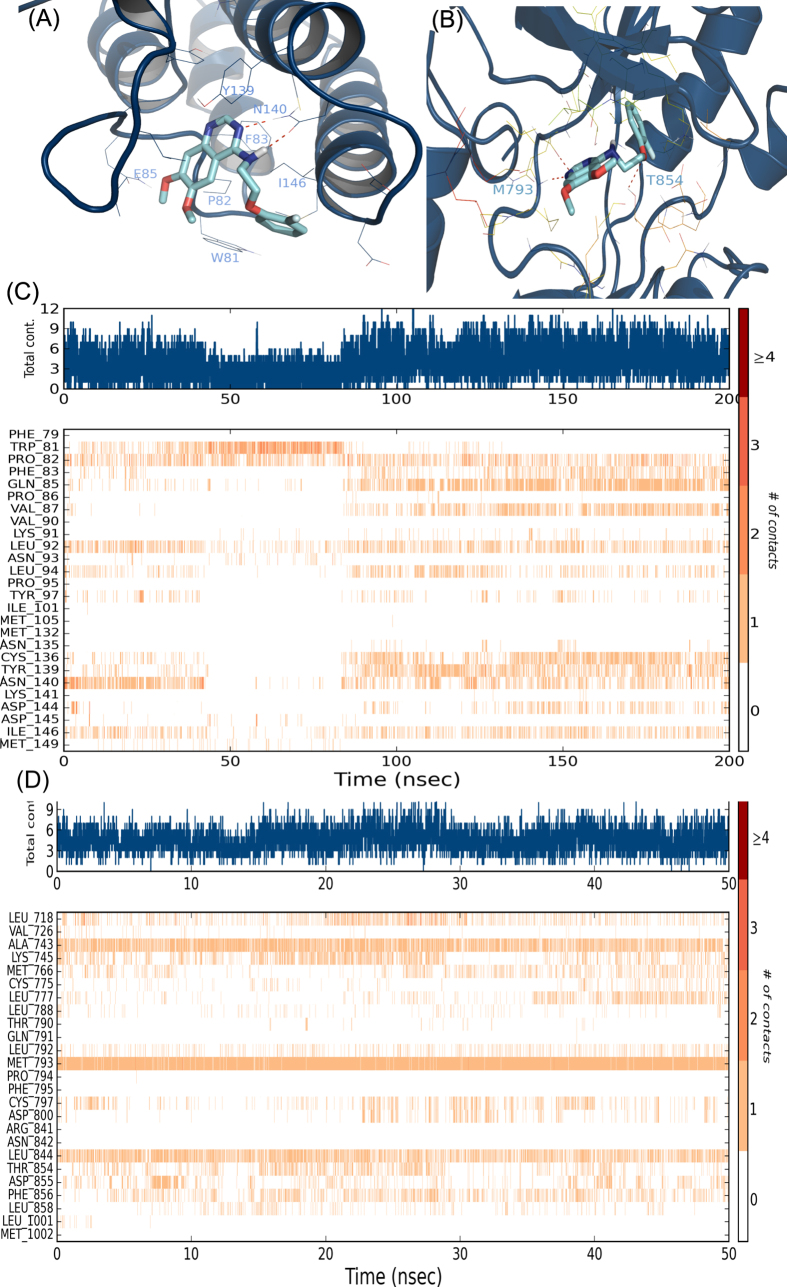
Molecular dynamics analysis Receptor ligand poses as obtained by docking simulations of 2870 in (A) BRD4(1) and (B) EGFR TKD and primary receptor ligand interactions (H-bonds, hydrophobic, ionic, water bridges) of 2870 for the duration of the MD simulations with (C) BRD4(1) and (D) EGFR TKD respectively. The top panel in (**C, D**) shows the total number of specific contacts the protein makes with the ligand over the course of the trajectory. The bottom panel shows which residues interact with the ligand in each trajectory over time. Some residues make more than one specific contact with the ligand, which is represented by a darker shade of orange, according to the scale to the right of the plot.

To characterize how 2870 binds to BRD4(1) at the atomic level and gain insights into binding dynamics, we performed a 200 nanosecond (ns) molecular dynamics (MD) simulation (see Methods). Simulation analysis showed a conserved interaction between pyrimidine nitrogen at position 6 and asparagine 140 (the conserved acetyl-lysine binding motif), proline 82, leucine 92 and isoleucine 146 (Fig. 8a). While this primary interaction was taking place, protein and ligand RMSD values stayed relatively low, indicating a stable binding conformation (Supplementary Figure 4a). However, from 40 to 80 ns, an increase in ligand RMSD is observed as the ligand switches its primary interaction to tryptophan 81 through aromatic stacking interactions, destabilizing the complex. After 80 ns, the compound returns to its original binding conformation and restabilizes the binding interactions observed previously throughout the remaining 120ns, with additional interactions observed with phenylalanine 83, glutamine 85, valine 87, cysteine 136 and tyrosine 139 (Fig. 8c).

Docking and MD were also performed with the EGFR tyrosine kinase domain (TKD) and compound 2870 to compare with the simulation results in BRD4. Two different crystal structures of the EGFR TKD from the PDB were used to build docking models (see Methods). The top docking score of 2870 in the TKD (−9.97) supports its observed high affinity. These results are also consistent with the MD simulation. Docking pose and MD results clearly show 2870 as a type I tyrosine kinase inhibitor due to its binding interactions with methionine 793 and threonine 854 in the DFG-in conformation of the kinase domain activation loop. MD results show stable RMSD values of 2870 throughout the entire 50ns simulation (Supplementary Figure 4b). Additional conserved residue interactions include alanine 743, lysine 745, cysteine 797, leucine 844, aspartic acid 855 and phenylalanine 856 (Fig. 8b,d). Throughout the time series 2870 interacted with M793, K745, T854 and D855 (all residues associated with ATP binding) 97, 26, 19 and 12% of the time.

## Discussion

Inhibition of cancer-promoting kinases is an established therapeutic strategy for the treatment of many cancers. Thirty kinase inhibitors are approved for use in humans and hundreds more are in clinical development^26^. Most of these drugs inhibit multiple kinases and in many cases their efficacy is related to some extent to their poly-pharmacology. Rational design of such “selectively unselective” kinase drugs that bind to the desired disease targets, but avoid off-target liabilities is very difficult due to the high similarity of the ATP binding site across the human kinome^27^. It is likely that most of the approved kinase drugs are marginally striking the balance favorably. In contrast, it may be easier to optimize multi-target kinase BET bromodomain inhibitors, and there is strong evidence that such compounds can exhibit favorable efficacy and pharmacology. For example, studies with ERBB2+ breast cancer have shown that targeting multiple alternative kinases upregulated by adaptive kinome reprogramming after the development of resistance to lapatinib increased growth inhibition of tumor cells variably. However, BET bromodomain inhibition in combination with EGFR kinase inhibition was the most effective at preventing kinome reprogramming due to its epigenetic regulation of multiple alternative kinases often necessary for resistance and propagating ERBB2+ cell growth^4^,^5^. It has also been suggested that kinase inhibitors may be privileged inhibitors of BET bromodomain proteins and there are several examples of well characterized potent dual bromodomain/kinase compounds, including a recent dual PLK1/BRD4 inhibitor^18^. As a proof of concept study to identify multi-target kinase BET bromodomain compounds we focused on EGFR TKD and BRD4(1) as a starting point for identifying more efficacious compounds targeting cancers containing an EGFR amplification. We further rationalized the presumed efficacy of a dual EGFR-BRD4 compound based on transcriptional response data from the LINCS project. Interestingly, 27 compounds from our docking study were also used in LINCS assays with many other docked compounds sharing high chemical similarity (Supplementary Figure 5).

Using an extensive computational screening pipeline we identified a first in class dual EGFR-BRD4 inhibitor, validated by several BRD4 and EGFR inhibition assays and further characterized by extensive molecular modeling. Our computational approach was based on ligand-based kinase classification models and BRD4 structure-based models integrated with physicochemical property predictors into a comprehensive virtual screening workflow. The kinase classifiers were trained on thousands of EGFR inhibitors against hundreds of thousands of kinase decoy compounds and the BRD4 structure-based models made use of several available co-crystal structures. To estimate EGFR activity we built Laplacien-modified Naïve Bayesian classifier ligand-based models, which had previously shown strong predictive performance. In addition, the method is scalable and applicable to large data sets, allows modeling in high-dimensional spaces while avoiding overfitting and is appropriate for structurally diverse dissimilar molecules, incorporating multiple activity classes into a single model (for example different binding modes). Laplacien-corrected Naïve Bayes classification is also reasonably resistant to noise such as false positives or false negatives. It should be noted however, that these are probabilistic models and performance is evaluated based on their ability to rank order compounds by a (predicted) property, which can be quantified by enrichment factor and ROC score (see Results). The statistics-based characterization of machine learning models (or any predictor) therefore should not be interpreted as a capability to predict any single active compound or a certain percentage of a small sample size. In addition, we observed compounds that were predicted active in the BRD4(1) ensemble docking model falling towards the bottom of the EstPGood cutoff that was used to prioritize compounds for EGFR activity using the ligand-based machine learning models. This is most likely the reason why only one of the BRD4 actives showed significant EGFR activity. It also suggests that we should be able to identify additional dual actives if we tested a larger number of top ranking compounds. Our high-throughput computational screening pipeline balanced predicted EGFR activity with favorable physicochemical properties before applying the BRD4(1) structure-based models. In this way we prioritized novel lead-like EGFR inhibitors among millions of compounds and then selecting the most likely BRD4(1) binders. This was a significant challenge, because we were looking at the intersection of two orthogonal predictions. Several known inhibitors of EGFR were included in our BRD4 consensus docking protocol, however, these compounds did not rank highly, supporting our focus on the discovery of novel compounds. We only tested for EGFR activity those compounds that we had already confirmed as BRD4(1) binders.

Although other groups had performed virtual screens to discover novel EGFR inhibitors^28^ as well as BRD4 interacting small molecules^29^, our approach is the first to look for multi-target dual inhibitors using a combination of ligand and structure-based models for each target. As a result of our computational pipeline, we ultimately selected and tested 24 compounds from over six million. We identified several novel BRD4 binders and one novel dual EGFR-BRD4 inhibitor, 2870, a first-in class compound. 2870 is a potent EGFR inhibitor (IC_50_: 44 nM) and a relatively weak ERBB2, ERBB4 and BRD4 inhibitor (IC_50_: 8.73, 24.2 and 9.02 *μ*M respectively). To better understand molecular binding interactions of 2870 in BRD4 and EGFR and to facilitate future rational optimization, we performed extensive all atom, explicit water MD simulations of 2870 in BRD4(1) and EGFR TKD. The 200 ns MD simulation of the predicted BRD4(1)-2870 complex appeared consistent with the more modest potency observed in the biochemical assays (compared to EGFR). The observed interactions throughout the duration of the simulation were important acetyl-lysine interaction motifs for BRD4(1). The initial docking pose shows primary interaction with asparagine 140, a conserved direct acetyl-lysine binding residue, through a direct hydrogen bond with the pyrimidine nitrogen at position 8 and 10 (Fig. 8a). Binding of 2870 appeared to switch between different interactions, but stabilize half way though the simulation with key interactions including asparagine 140, the chief acetyl-lysine binding residue, and isoleucine 146, characterized as the “gatekeeper” residue for binding of BRD4 to acetylated histones, indicating that the molecule keeps the residue in an occupied conformation (Fig. 8c). Structural analysis of BRD4(1) at atomic resolution indicates that isoleucine 146 adopts a closed conformation in the absence of binding^15^. 2870 primarily interacts at the BC loop towards helix C. Its 2-aryloxyethylamino moiety off the quinazoline scaffold is partially solvent exposed. Similarly the methoxy substituents do not appear to have a strong interaction in the binding site.

In contrast to BRD4(1), 2870 binds rigidly in the ATP binding region of EGFR. The binding orientation is the same as Lapatinib, Erlotinib, and other 4-amino-quanzoline inhibitors (Supplementary Figure 6). The quinazoline scaffold binds to the hinge loop and the 2-aryloxyethylamino-4-quinazoline substituent directed towards the kinase gatekeeper residue and C helix; the alkoxy moieties towards the D helix adjacent to the hinge loop (Fig. 8b,d). Therefore, 2870 is a type I kinase inhibitor binding in the DFG-in conformation of the activation loop.

Interestingly, the 4-amino-quinazoline motif interacts with BRD4 Asn 140 and the EGFR hinge region, thereby rationalizing its dual activity. 2870 binds EGFR with an IC_50_ of 0.044 *μ*M as compared to 9.02 *μ*M for BRD4. This difference in binding affinity can be rationalized based on the trajectories obtained from the MD simulations of 2870 for each target. The molecular interactions analyzed in the MD simulations suggest possible positions for chemical optimization of 2870 to develop derivatives with more equal BRD4 and EGFR potency, which would likely increase compound efficacy. These include the alkoxy residues and the 4-alkyl-/aryl-amino substituents. Although the 4-amino-quanzoline is a well-known EGFR scaffold, all drugs have a 4-aryl substituent, in contrast to 2870, which is alkyl-amino substituted (2-fluorophenoxy-2-ethyl-amino). This allows more flexibility, but still enables the formation of aromatic interactions in the kinase binding site, and may explain its dual EGFR-BRD4 activity. This significant difference is also reflected in its low Tanimoto similarity to known EGFR kinase drugs. Among known EGFR inhibitors, 2870 is least dissimilar to Erlotinib (0.446), compared to Lapatinib (0.389), Gefitinib (0.261), and AEE788 (0.239). 2870 shares even less chemical similarity to any known BRD4(1) binder with the closest compound being 0.3 similar. 2870 can therefore be considered a novel multi-target inhibitor with respect to both BRD4 and EGFR.

Our results do not support or reject the hypothesis that known kinase inhibitors may be privileged BET binders. We are currently performing analyses to investigate this further. In our approach we have demonstrated that it is possible to computationally develop such dual inhibitor compounds. We are currently extending our screening approach to study other important target combinations and we believe that our in-silico approach can be generalized to discover a variety of novel multitarget kinase BET inhibitors and chemotypes.

Novel compounds with specific polypharmacology have great potential as cancer therapeutics with increased clinical efficacy. Orthogonal multitargeted therapies are needed to counteract compensatory mechanisms present in tumor cell populations, such as the adaptive bypass response, which often leads to drug resistance and clonal evolution of the cancer. Single multi-target compounds have advantages over combination therapies, including reduced risk of drug-drug interaction and toxicity, improved efficacy, regulatory approval and intellectual property. Here we demonstrated a proof of concept study implementing a pipeline to identify dual EGFR and BRD4 inhibitors. We discovered the first in class dual multitarget EGFR and BRD4 inhibitor and predict that many dual kinase-BET inhibitors can be identified using this approach. We hope this will contribute to developing novel clinical drug leads for the treatment of cancers resistant to current treatment regimens.

## Methods

### Analysis of transcriptional response signatures

LINCS L1000 transcriptional response signatures were generated at the Broad institute and are available via LINCSCloud (http://www.lincscloud.org/) and via the BD2K LINCS Data Coordination and Integration Center (http://bd2k-lincs.org/). We had previously processed all LINCS signatures in the LINCS Information FrameWork (LIFE, http://life.ccs.miami.edu/) and only used L1000 signatures with the annotation “is gold”, indicating the highest quality of aggregate data. Data was analyzed by cell lines, compounds and other metadata and the largest available consistent dataset was selected, namely MCF7 cells at 10 *μ*M compound concentration after 24 hours. All pairwise Pearson correlation coefficients were computed from the L1000 z-score signatures for all 2482 drug perturbations. Data were processed and reformatted using Pipeline Pilot 8.0 (Accelrys) components. Small molecule perturbagens were annotated with their known targets extracted from the ChEMBL 20 database after mapping LINCS compounds to ChEMBL IDs. These mappings are now also available via UniChem (https://www.ebi.ac.uk/unichem/). Twelve EGFR inhibitors and four BRD4 binders were identified. They are all well annotated, known nanomolar activity compounds (Supplementary Table 1). Welch two sample t-test was performed to compare the population of EGFR inhibitors, BRD4 binders, the intersection of BRD4 and EGFR inhibitors (i.e. all pairs of BRD4 and EGFR, but not BRD4-BRD4 or EGFR-EGFR compounds) and all Other compounds based on their signature Pearson correlation coefficients (R^2^) using the program R (http://www.r-project.org/). Significant p-values in the range of 10^−5^ to 10^−3^ were obtained when comparing EGFR to Other, EGFR to BRD4, BRD4 to Other as well as the intersection of EGFR-BRD4 to Other (Fig. 1). Lastly, after the structure based modeling was completed, all predicted compounds used in our docking study were compared to compounds used in LINCS assays by Tanimoto chemical similarity using Pipeline Pilot (Supplementary Figure 5). 27 compounds used in our docking study have been used in LINCS assays with most other compounds sharing high chemical similarity.

### Kinase machine learning classification models

Laplacien-modified Naïve Bayesian classifiers using topological fingerprints were built and validated as previously described^21^. However, by contrast to our initial report, we used over twice the amount of data to train the classifiers; the Q2 2013 data release of the Kinase Knowledge Base (KKB; Eidogen-Sertanty; http://eidogen.com/) was standardized and processed in the same manner as described. Using extended connectivity fingerprints (ECFP4), two models for EGFR were built based on a total of 591,744 unique kinase compounds: one with 3,058 actives defined as pIC_50_/pK_*i*_ ≥ 7 and another with 4,785 actives of pIC_50_/pK_*i*_ ≥ 6. Both models have excellent ROC scores of 0.98 to 0.99 based on 50/50 training/test set and estimated based on the leave-one-out cross validations (Supplementary Figure 1). The enrichment factors among 1% of the dataset are 78 and 66, respectively. This is close to the upper bound (100) for the given subset (1%) and the ratio of actives versus total compounds.

### BRD4 docking models

We used the Schrodinger 2014.2 software suite for structure-based manipulations and simulations. Seven BRD4 co-crystal structures were selected from the Protein Data Bank (PDB) from 65 available structures (January 2014). Three crystal structures for the extensively characterized BRD4 inhibitors namely JQ1 (3MXF), I-BET151 (3ZYU) and I-BET762 (3P5O) were chosen. Other PDB crystal structures included 4HXS, 4C67, 4LR6 and 4LYW. Overall, the selection criteria included the atomic resolution of the X-ray crystal structure, diversity in the co-crystal ligand scaffold and ligand interactions in the binding site. From the seven crystal structures chosen, we identified conserved waters found in/around the ligand binding sites, in order to incorporate likely protein-ligand water bridges into our models. To do this we aligned all BRD4 crystal structures and determined which waters fell within 5 Å of the active site. We then kept these waters specifically in the preparation process of two out of the seven crystal structures, in addition to those that we had already prepared. This amounted to nine prepared crystal structures with which to produce docking models.

BRD4 protein structures were pre-processed using the protein preparation workflow in Maestro 9.5 to assign bond orders and refine the structure including hydrogen bond optimization and constrained minimization. Where needed, missing side chains were added using Schrodinger Prime. For each structure, one protein chain with the co-crystal ligand was kept, and water molecules were deleted beyond 5 Å from heteroatom groups. In addition, the internal hydrogen bond network was optimized, followed by constrained energy minimization. For the resulting nine structure representations, docking grids were generated around the co-crystal binding sites using Schrodinger Glide in the default settings. Re-docking each co-crystal ligand into its corresponding structure validated models; in each case the co-crystal pose was reproduced with RMSD values < 1 Å

### BRD4 small molecule activity data and decoy datasets

The activity data for the known BRD4 inhibitors was extracted from the ChEMBL18 database (May 7, 2014) and in addition curated from the recent literature^18^,^19^,^29^,^30^,^31^,^32^,^33^,^34^,^35^. Only the compounds with activity data (IC_50_/K_*d*_/K_*i*_) were used for further processing. Data were combined and the duplicates were removed. Chemical structures were standardized including protonation states, canonical tautomer, salt removal and proper stereochemical and geometric configuration using Pipeline Pilot 8.0 (Accelrys) and unique IDs were assigned. Activity data was p-transformed (–Log_10_) and aggregated by unique compounds using the median activity value for compounds that had multiple activity values available.

To estimate enrichment and determine thresholds for docking score significance, the 246 unique BRD4 inhibitors were submitted for generating the decoy sets from the Directory of Useful Decoys, Enhanced (DUD-E)^36^. This generated 50–100 decoy compounds per submitted ligand SMILES. The 246 original ligands and the corresponding 15,250 decoys compounds (15,496 compounds total) were then pre-processed for docking.

### Ensemble docking protocol and data fusion

#### Ligand Preparation

Ionization states, tautomeric forms, and 3D conformations for 15,496 SMILES (246 known BRD4 ligands and 15,250 decoy compounds) were generated using LigPrep (Schrodinger 2014.2). The default conditions were maintained; except for the ionization states which were generated at the target pH of 7 ± 2 using Epik including the original state. The resulting 3D ligand representations were exported as structure data files and unique IDs were assigned based on unique canonical structures to facilitate post-docking hierarchical data fusion.

#### Docking protocol

Ligand representations obtained via LigPrep for all (BRD4 known active and decoy) compounds were docked against all 9 prepared BRD4(1) models using Glide (Schrodinger 2014.2) in standard precision (SP) with the default settings, except writing out at most 5 poses per ligand representation and including 25 poses per ligand for post-docking minimization. All docking results were exported from Maestro 9.5 as delimited text files. The docking scores were aggregated hierarchically at three levels, at each level generating average and top docking scores as follows: (i) for each unique ligand representation structure, the scores of all corresponding docking poses were aggregated; (ii) pose-aggregate scores of unique ligand representations (generated in LigPrep) were aggregated by unique (original) compound structure (across ionization states and tautomers); (iii) aggregate compound scores were combined across all protein structures. At the ligand representation level, we considered top and average top 3 and average top 5 pose scores, at the compound level, the top and average of all corresponding ligand representations, and at the protein level, the top and average top 3 and top 5 scores, giving a total of 18 aggregation methods (Supplementary Figure 2). Based on model evaluation results for all aggregation methods, for the final results we used the top scores obtained across all levels; for each ligand structure this corresponds to the best pose for the best ligand representation in the best protein-docking model.

#### Evaluation and characterization of predictions

The docking method was evaluated by the receiver operating characteristic (ROC), enrichment factors (EF) and by correlation of aggregate docking scores and activity data using the known BRD4 inhibitors and the large decoy dataset (described above). The aggregate docking scores (BRD4_−_GlideSP_−_Top1_−_Top_−_Top1) exhibited the best overall relation to reported activity. The obtained ROC curves are shown in Fig. 2. The sensitivity, specificity, and accuracy for different activity and docking score cutoffs and EF at 0.1%, 1% and 20% subsets are shown in (Table 1). Sensitivity (S) is defined as true positive rate (TPR), specificity (SP) is true negative rate (TNR) and accuracy is the overall correct prediction rate [(TP + TN)/N]. The ROC is [S/(1-SP)], i.e. TPR over FPR. EF is [TP(subset)/n(subset)]/[P(total)/n(total)], i.e. the ratio of true positives detected in the subset divided by the fraction of overall (total) positives. The known BRD4 inhibitors were clustered by maximum common substructure using ChemAxon JCluster (MultiMCS)^37^,^38^ and also the partitioning algorithm with topological Fingerprints as implemented in Pipeline Pilot 8.0. Correlation coefficients were computed for aggregate docking scores versus median activity for all clusters.

### Virtual screening workflow to identify dual EGFR-BRD4 inhibitors

EGFR Laplacien-modified Naïve Baysian classification models were applied to the eMolecules (http://emolecules.com/) database of over 6 million commercially available compounds (downloaded in December 2013), which were standardized in the same manner as the small molecule kinase inhibitor model training sets, specifically removing salts and other addends, standardizing stereoisomers and geometric isomers, generating unique tautomers, physiological ionization states and creating canonical SMILES representations. Physicochemical properties were calculated for all compounds using Pipeline Pilot 8.0 (Accelrys). For the selection of physicochemically favorable compounds, we only considered those predicted as EGFR active (after minimizing false positives and false negatives) in the both the EGFR (pIC_50_/pK_*i*_ ≥ 6) and EGFR (pIC_50_/pK_*i*_ ≥ 7 models). To sample most suitable compounds, we selected compounds with very high predicted probability of EGFR activity (EstPGood) and less stringent physicochemical property criteria, but also compounds with relaxed EstPGood cutoffs and more strict physicochemical property filters. Overall, the following parameters were used: EGFR (pIC_50_/pK_*i*_ ≥ 6 predicted probability (EstPGood) ≥ 0.05; molecular weight ≤500; hydrogen bond acceptors ≤ 10; hydrogen bond donors ≤ 5; rotatable bonds ≤ 10; molecular polar solvent accessible surface area ≤250; ALogP ≤5. These criteria yielded 908 compounds. Ligand preparation was performed on all 908 compounds, creating 2,477 ligand representations to be used for docking. Docking was performed using all 9 models followed by the aggregation/data fusion of docking scores and analysis using TIBCO Spotfire. Based on model evaluation (enrichment) using known actives and large decoy sets (as described above), statistical thresholds to select likely actives were established at z-scores of −2 and −3 of the predicted active docked compound distribution. After rank ordering compounds, additional selection criteria included EstPGood of the EGFR classifiers, commercial provider availability, physicochemical properties and chemical diversity. Upon final manual review and selection among 108 compounds, 47 of the compounds were found commercially available from the vendor Enamine, LLC, 25 were selected and 24 were ultimately purchased and received.

### EGFR structure-based models and docking

Protein preparation, docking protocol and model validation for EGFR were performed in the same manner as for BRD4 described above. Two crystal structures (1XKK and 1M17) were chosen from 132 available based on the highest chemical similarity of their co-crystal ligand to the dual EGFR-BRD4 compound 2870. Three other co-crystal ligands were also used in the docking study along with 2870 to confirm reproducibility of known ligand binding poses.

### Molecular dynamics simulations

All atom (OPLS force field) explicit water molecular dynamics simulations were performed using the Desmond 2014.2 software suite via Maestro 9.8^39^. The Desmond software automatically sets up the systems (adjust charges, adds water molecules) and performs several rounds of minimization and short simulations before the production runs. Molecular dynamics (MD) was run on the Pegasus 2 cluster from the Center for Computational Science at the University of Miami (http://ccs.miami.edu/hpc/) using 48 processors and completed in 53 hours for the 50ns EGFR simulation and 198 hours for the 200ns BRD4 simulation. Simulation analysis was performed using the Desmond trajectory analysis software.

### BRD4(1) biochemical alphascreen assay

The BRD4(1) alpha screen assay was performed with a pre-acetylated Biotin-Histone 4 Peptide [H4K5/8/12/16(Ac)] 1 – 21 (AnaSpec #64989-025, 0.25 mg). A 200 *μ*M stock was made and stored at −80 °C. BRD4-BD1 (49–170, BPS Bio #31042) was stored at −80 °C at a concentration of 50 *μ*M. All compounds used in assays were kept in a 10 mM stock at −80 °C, with I-BET151 being used as a control. The assay buffer contained 50 mM HEPES, 100 mM NaCl, and 0.1% BSA at a pH of 7.4, and was stored at 4 °C. 10% CHAPS (Sigma C3023) was added to the assay buffer before each experiment and was stored at room temperature.

Before use, assay buffer was equilibrated to room temperature and supplemented with 0.05% CHAPS. 3X His-BRD4-BD1 was diluted in assay buffer to 600 nM with a final concentration of 200 nM in a 12 *μ*l reaction volume. 4 *μ*l per well of 3X His-BRD4-BD1 was added to an OptiPlate-384 (Perkin Elmer #6007290) covered with Top Seal-A Film (Perkin Elmer #6005250) and pulsed at 900 × g. 3X BRD Inhibitors or DMSO vehicle was prepared in Assay Buffer starting at 300 *μ*M and diluting 3X to 46 nM. 4 *μ*l of buffer (positive control), 3X DMSO Vehicle, or 3X BRD Inhibitor (I-BET151 as control) were added to each well, sealed with Top Seal-A film and pulsed at 900 × g. The plate was then incubated at room temperature for 30 minutes. 3X Biotin-H4K4Ac was prepared by diluting stock in Assay Buffer to 600 nM, with a final concentration of 200 nM in a 12 *μ*l reaction volume. 4 *μ*l of Biotin-H4K4Ac was added per well, sealed and pulsed at 900 × g. The plate was then incubated at room temperature for another 30 minutes. AlphaLISA Nickel Chelate Acceptor Beads (Perkin Elmer #1547780, 5 mg/ml, 4 °C) and AlphaScreen Streptavidin Donor Beads (Perkin Elmer #6760002, 5 mg/ml, 4 °C) were prepared at 2.5X by diluting in Assay Buffer to 25 *μ*g/ml with a final concentration of each bead equaling 10 *μ*g/ml in a 20 *μ*l reaction volume. 8 *μ*l per well of Donor and Acceptor beads were added in the dark to each well, sealed, wrapped with foil to protect from light, and pulsed at 900 × g. The plate was then incubated at room temperature for 60 minutes and read on an EnVision instrument using Alpha Mode (680 excitiation/615 emission/read signal). Dose response curves were obtained and interpreted using GraphPad Prism software for curve fitting to obtain IC_50_ values. To confirm results, a Perkin Elmer Tru-Hits counter screen was performed with the same assay system to determine if any compounds were interacting with the assay components, allowing for the determination of false positives.

### Differential scanning fluorimetry

The inhibitory activity of compound Z118332870 (2870) against BRD4(1) was assessed by DSF using a StepOnePlus Real-Time PCR system (Applied Biosystems). Purified BRD4(1) (4 *μ*M final concentration; 10 mM HEPES (pH 7.5), 100 mM NaCl, and 1 mM DTT) was assayed, in triplicates, in a 96-well plate. Compound was added to a final concentration of 100 *μ*M and 2% DMSO. Protein Thermal Shift Dye (1:8000; Applied Biosystems) was used as the fluorescent probe and fluorescence was measured using the ROX Reporter channel (620 nm). Protein stability was investigated by programing the thermocycler to increase the temperature from 25 °C to 99 °C using 0.2 °C increments and 10 second incubations per increment. The inflection point of the transition curve/melting temperature (T_*m*_) was calculated using the Boltzmann equation within the Protein Thermal Shift Software (v.1.1) (Applied Biosystems). The ΔT_*m*_ was calculated by using DMSO control wells as a reference.

### EGFR kinase activity enzyme assay

A radioisotope filter binding method was used in the EGFR kinase hotspot assay, performed by the Reaction Biology Corporation, LLC. The base reaction buffer was composed of 20 mM HEPES (pH 7.5); 10 mM MgCl_2_, 1 mM EGTA, 0.02% Brij35, 0.02 mg/mL BSA, 0.1 mM Na_3_VO_4_, 2 mM DTT, and 1% DMSO. The substrate was freshly prepared using the base reaction buffer. Kinase was added into the substrate solution at a concentration of 10 *μ*M and gently mixed. Compounds were then added into the kinase reaction mixture. At this point, the ^33^P-ATP was added into the reaction mixture to initiate the reaction. The reaction was incubated for 120 minutes at room temperature. After completion, reactions were spotted onto P81 ion exchange paper (Whatman #3698-915). Filters were then washed extensively in 0.75% Phosphoric acid and a scintillation counter was used to quantify the amount of radioactivity on the filter.

## Additional Information

**How to cite this article**: Allen, B. K. *et al.* Large-Scale Computational Screening Identifies First in Class Multitarget Inhibitor of EGFR Kinase and BRD4. *Sci. Rep.* **5**, 16924; doi: 10.1038/srep16924 (2015).

## Supplementary Material

Supplementary Information

## Figures and Tables

**Table 1:** Evaluation and characterization of the BRD4 docking data fusion model.

ActivityCutoff	Docking ScoreCutoff	S	SPC	ACC	ROCScore*	EF_0.1_	EF_1_	EF_20_
5	−7	0.96	0.65	0.66	0.929	59.4	34.2	4.4
5	−8	0.60	0.96	0.95				
6	−7	0.95	0.65	0.65	0.927	101.5	50.3	4.3
6	−8	0.68	0.95	0.95				

For different activity and docking score cutoffs, sensitivity (S), specificity (SPC), accuracy (ACC), ROC scores and enrichment factors at 0.1, 1 and 20 percent screened subset are shown. (*) To compute the ROC score, docking scores were used for rank ordering without a cutoff.

**Table 2:** Binding affinity of 2870.

2870	
Target	IC_50_ (μM)
BRD4(1)	9.02
EGFR (ERBB1)	0.044
ERBB2	8.73
ERBB4	24.2

Activity of compound 2870 against BRD4(1) by alpha screen and EGFR TKD and other related kinase family members obtained by kinase activity enzyme profiling.
