# Supplementary material for: Large-Scale Computational Screening Identifies First in Class Multitarget Inhibitor of EGFR Kinase and BRD4
Source: Sci Rep. 2015 Nov 24;5:16924. doi: 10.1038/srep16924 (PMC4657038; doi:10.1038/srep16924)
Supplement: Supplementary Information [file srep16924-s1.doc]

Supplementary Information – Allen, B.K. et al. 2015

**Large-Scale Computational Screening Identifies First in Class Multitarget Inhibitor of EGFR Kinase and BRD4**

Bryce K. Allen^1,2,5,6^, Saurabh Mehta^1,2,3^, Stewart Ember^4^, Ernst W. J. Schonbrunn^4^, Nagi Ayad^5,6,*^, Stephan C. Schürer^1,2,5,*^

1. Department of Molecular and Cellular Pharmacology, Miller school of Medicine, University of Miami, Miami, FL, US.

2. Center for Computational Sciences, University of Miami, Miami, FL, US.

3. Department of Applied Chemistry, Delhi Technological University, Delhi, India.

4. Drug Discovery Department, H. Lee Moffitt Cancer Center and Research Institute, Tampa, FL, US.

5. Center for Therapeutic Innovation, Miller School of Medicine, University of Miami, Miami, FL, US.

6. Miami Project to Cure Paralysis, Department of Psychiatry and Behavioral Sciences, Miller School of Medicine, University of Miami, Miami, FL, US.

* To whom correspondence should be addressed: [sschurer@miami.edu](mailto:sschurer@miami.edu); nayad@miami.edu.

**Supporting Material**

**Supplementary Table 1.** Twelve known EGFR and four known BRD4 inhibitors were used in our LINCS transcriptional profile correlation study. Structures of compounds are shown with corresponding LINCS IDs (SM_LINCS_ID), PubChem CIDs (SM_PUBCHEM_CID) and target annotations.
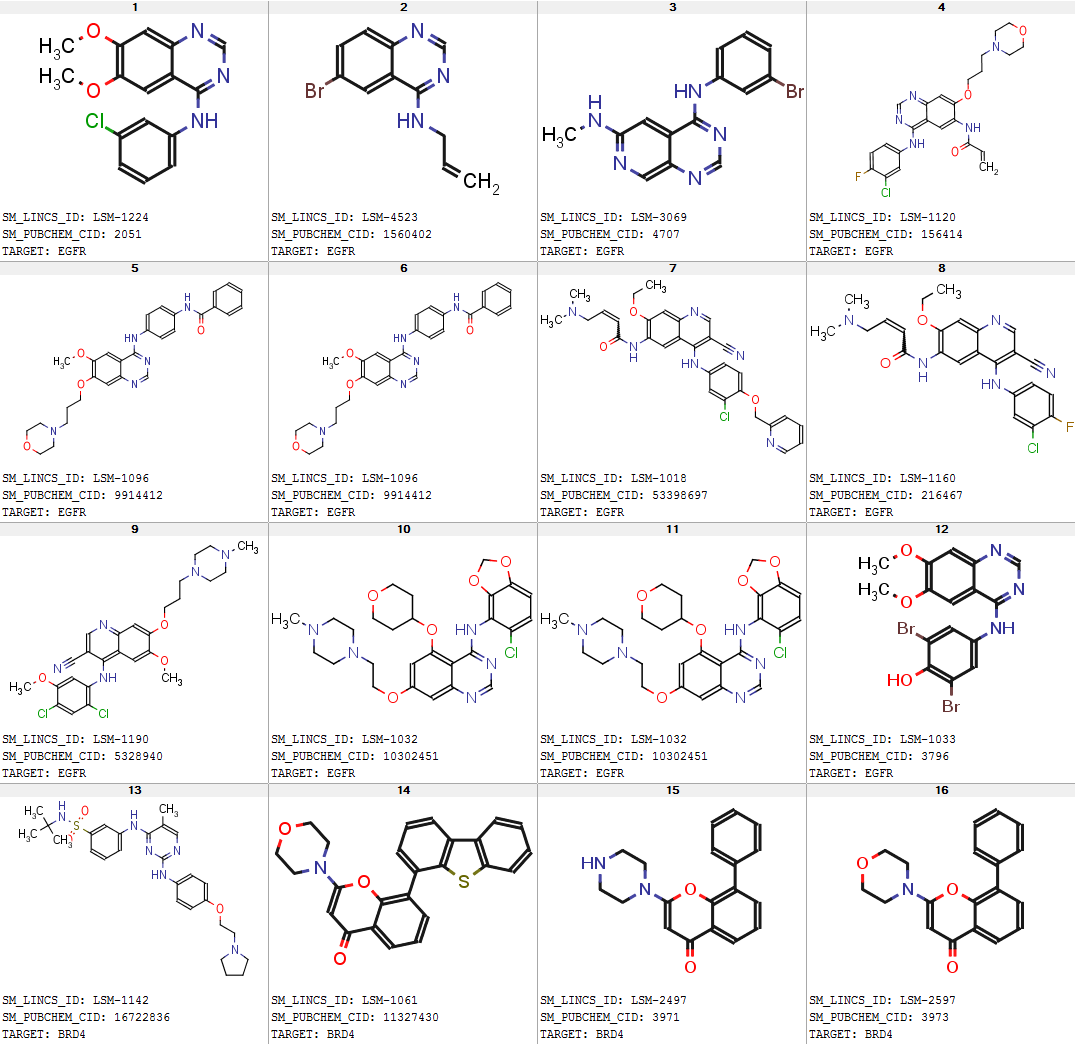


**Supplementary Figure 1.** Receiver operating characteristic (ROC) curves for 50% of the EGFR kinase model dataset at two different activity cutoffs (pIC50 ≥ 6 & 7). 20 cross validation runs were averaged with randomly selected 50/50 split training/test compounds. The ROC score of A) pIC50 ≥ 6 was 0.99 and B) pIC50 ≥ 7 was 0.98.


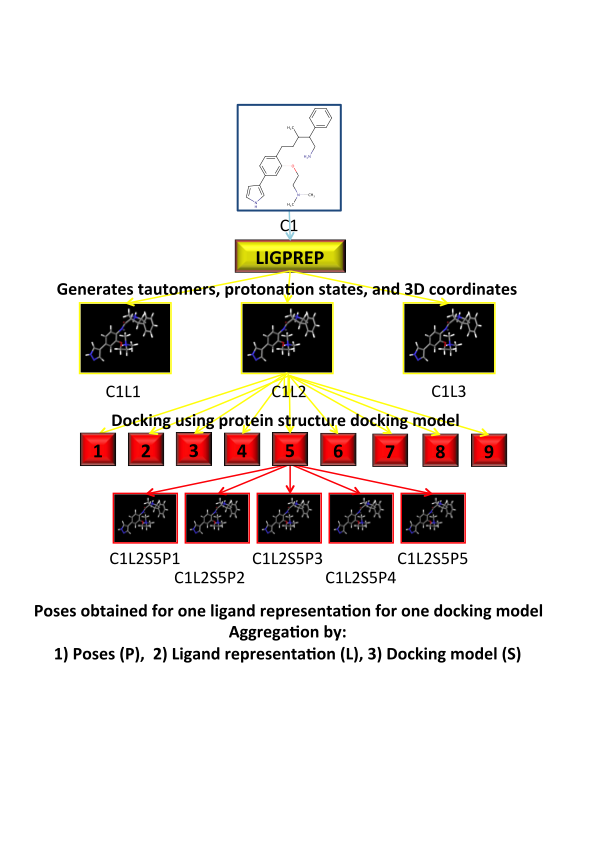


**Supplementary Figure 2.** Aggregation of results for the ensemble docking data fusion model. Starting with a compound C1, ligand representations (C1L1, C1L2, C2L3, etc.) are generated using the LigPrep module in Schrodinger Maestro. Each of these representations is docked against nine docking models (protein structure) and for each model the top 5 poses are kept and scored. For example, if C1L2 is docked against protein structure docking model S5, we would obtain C1L2S5P1 – C1L2S5P5 (compound 1, ligand representation 2, structure model 5, poses 1 to 5). We investigated various options of aggregating the results across the three levels (pose, ligand representation and structure model) to maximize the performance of the overall ensemble data fusion model using the test dataset. Specifically, results were aggregated by pose for each ligand representation investigating the top, the average of the top three and the average of the top five poses. Secondly, each of these would be aggregated across the ligand representations, by ether taking the best scoring ligand representation or the average of all ligand representations. These aggregations are referred to as the Top1-Top, Top3-Top, Top5-Top, Top1-Avg, Top3-Avg, and Top5-Avg. Finally each of these scores is then aggregated across the protein structures used for docking (docking model), again keeping the best or the average of the best three or best five; these are similarly referred to as -Top1, -Top3 and -Top5. Based on our enrichment analyses we chose the Top1-Top-Top1 aggregation method for the BRD4(1) docking data. However, there may be applications when using averages may be better suited.

**Supplementary Table 2.** 24 prioritized compounds commercially available from Enamine, LLC. Molecular structures, molecular weight, chemical formula and Enamine ID are shown.


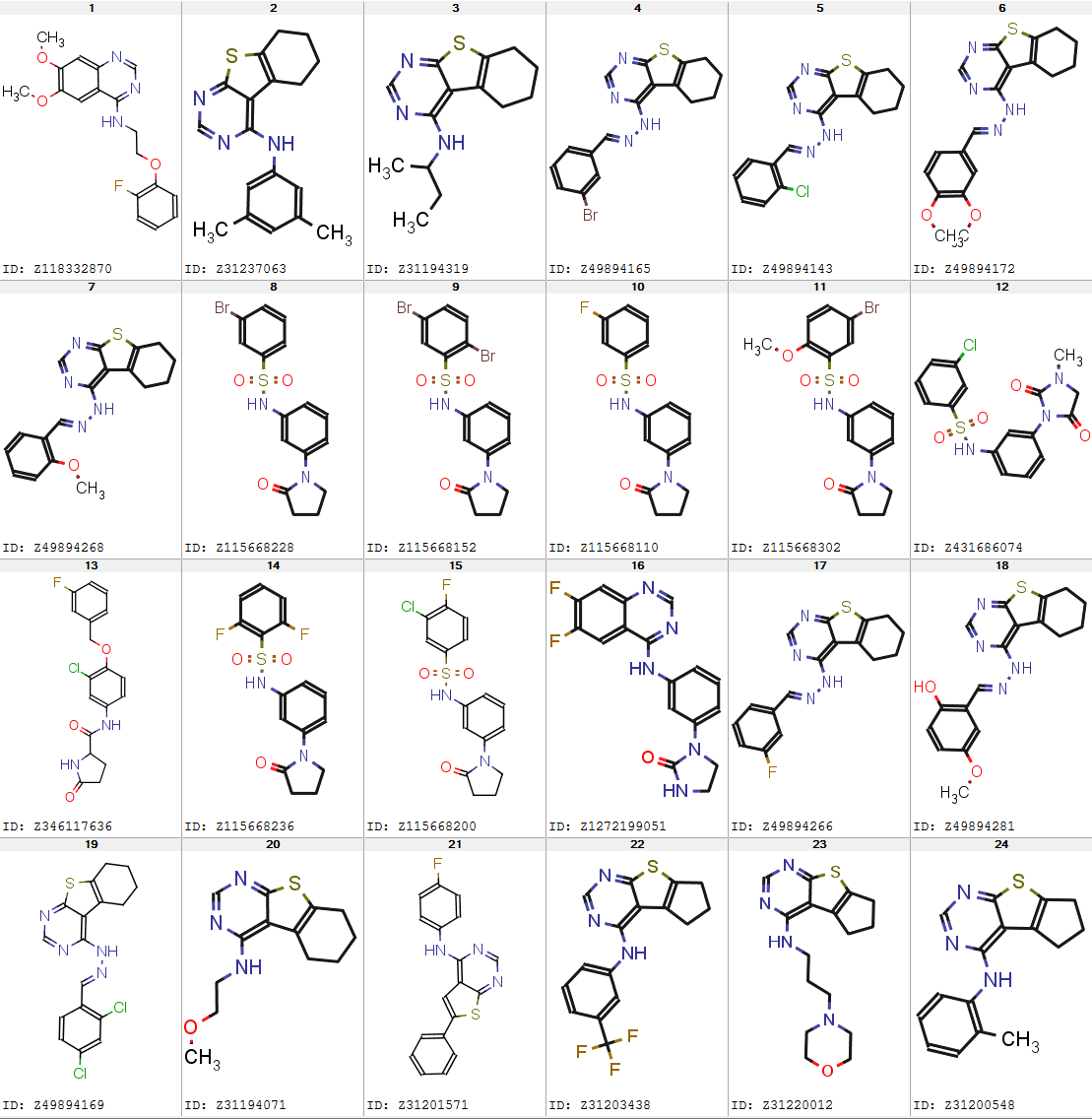


**Supplementary Figure 3.** Bar graph of counter screening results for compound 2870 using the Perkin Elmer Tru-Hits assay. Biotin was used as a negative control to keep beads from binding to each other, prohibiting emission. Compound 2870 performed equally well as DMSO, when normalized to assay buffer, N = 3.


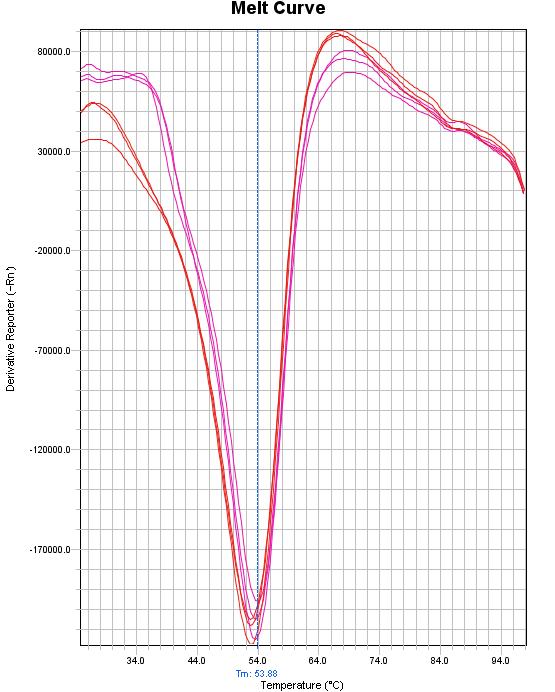


**Supplementary Figure 4.** The inhibitory activity of compound 2870 against BRD4(1) was assessed by differential scanning flourimitry (DSF) in two independent experiments (N=3 per experiment) using a StepOnePlus Real-Time PCR system. Melting temperature curves from the first experiment in the presence of DMSO (red) and 2870 (magenta) are shown above. The dotted line shows the Tm value for 2870. The mean ΔTm was calculated to be 1.15°C relative to DMSO.


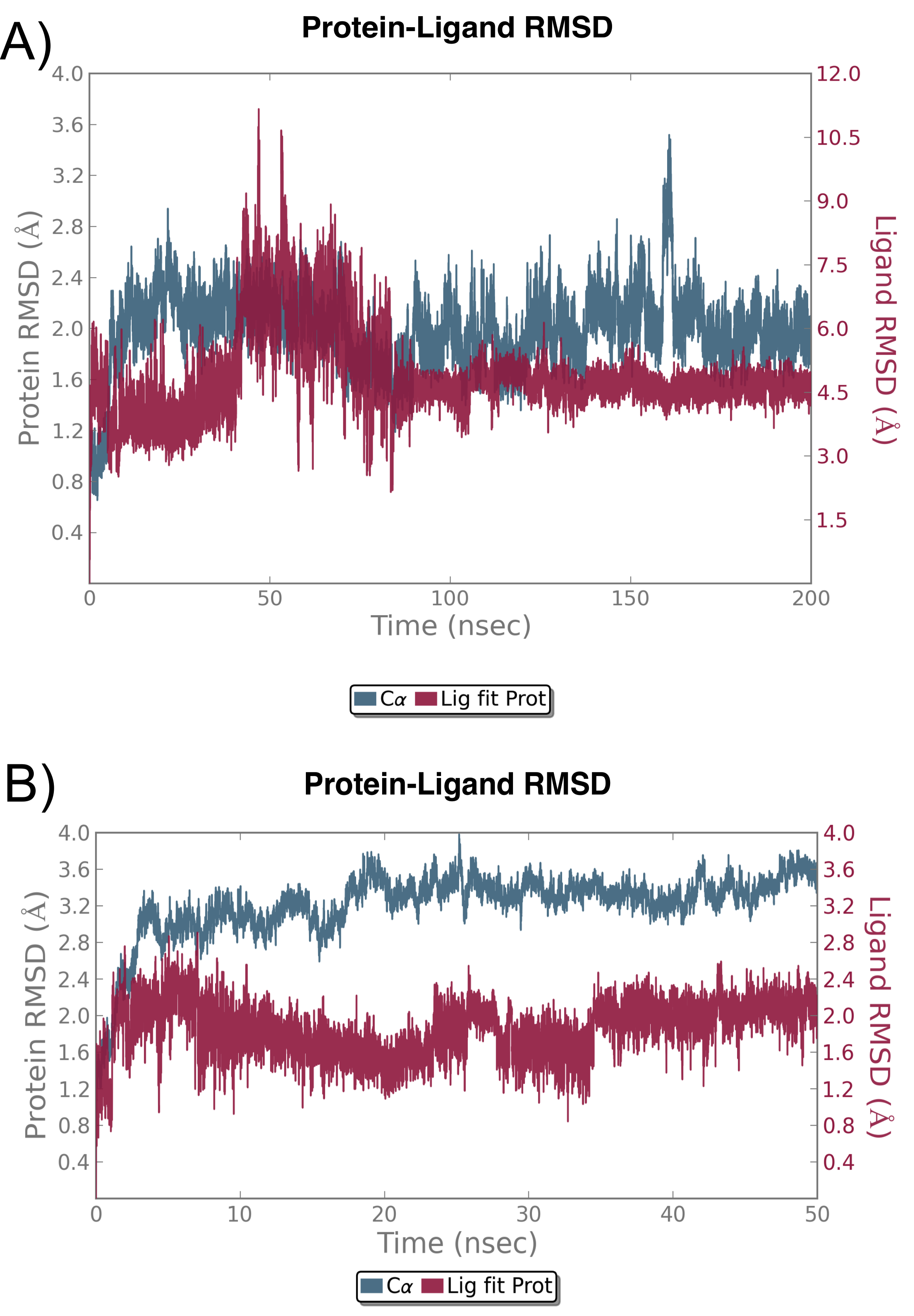


**Supplementary Figure 5.** RMSD evolution of the protein based on C-alpha carbon atoms (left Y-axis) and the ligand based on al heavy atoms (right Y axis) for the duration of the MD simulations. All protein frames are first aligned on the reference frame backbone, and then the RMSD is calculated. Ligand RMSD indicates how stable the ligand is with respect to the protein and its binding pocket. In the above plot ‘Lig fit Prot’ shows the RMSD of a ligand when the protein-ligand complex is first aligned on the protein backbone of the reference, and then the RMSD of the ligand heavy atoms is measured. A) RMSD results from the BRD4 and B) EGFR molecular dynamics simulation.

**Supplementary Figure 6.** A) BRD4(1) docking score on the x-axis and EGFR EstPGood for pVal6 on the y-axis. Large circles represent compounds that have been included and tested in LINCS assays. The color of the circles represents their chemical similarity to LINCS compounds with darker circles being more chemically similar than lighter circles. B) A histogram showing the distribution of compounds chemically similar to LINCS compounds used in our BRD4 docking set. The colors of the columns represent the average EGFR EstPGood score for pVal6 with darker columns representing higher average predicted activity against EGFR than lighter columns. Many of the LINCS compounds are known kinase inhibitors.


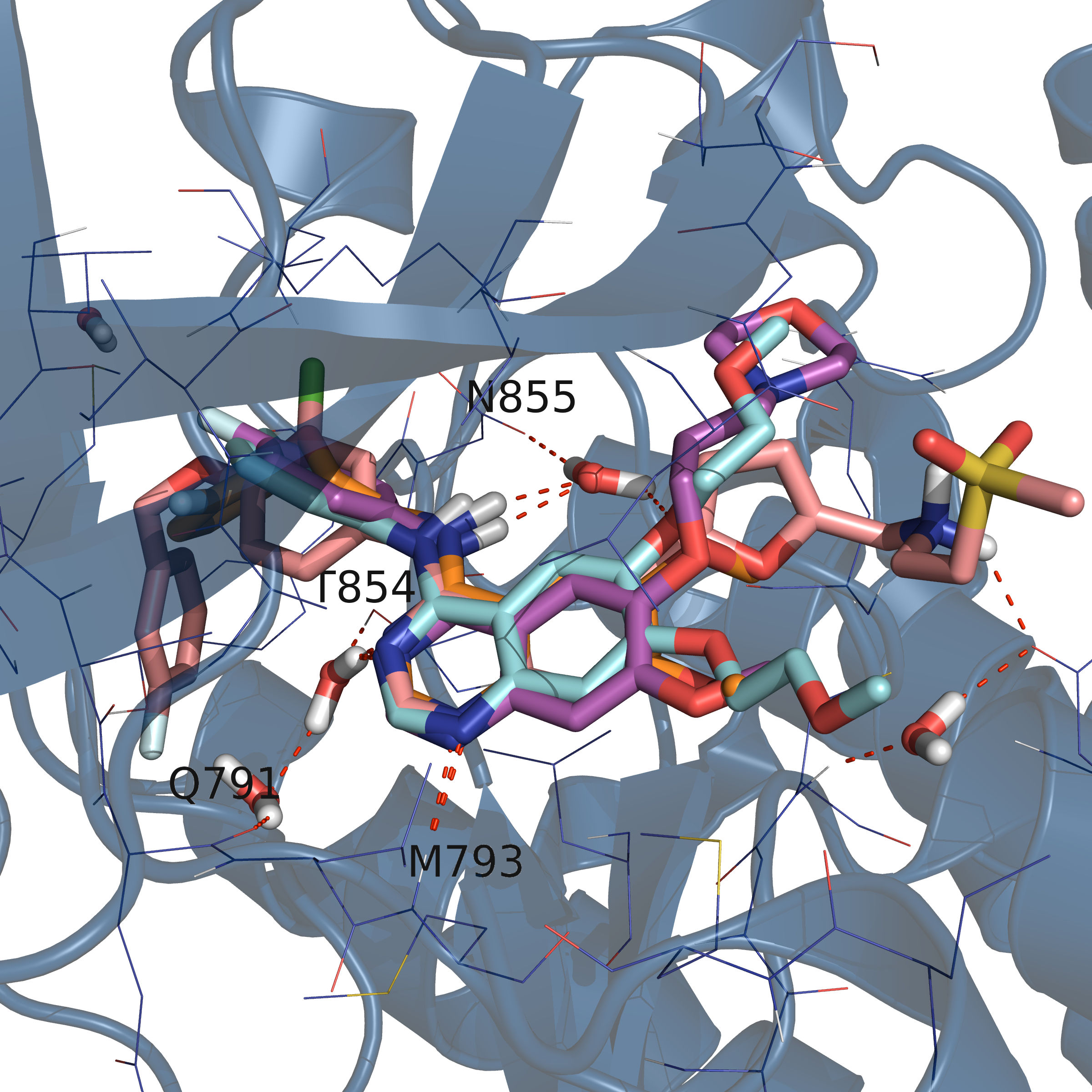


**Supplementary Figure 7.**  Binding poses of 2870 (purple), Erlotinib (teal), Lapatinib (peach) and Gefitinib (orange) docking into the EGFR kinase domain (prepared from PDB 1XKK). Conserved binding interactions are seen with methionine 793 (hinge loop), threonine 854 and asparagine 855, as well as some interactions with glutamine 791.
